# A closer look at the stoma: multimodal imaging of patients with ileostomies and colostomies

**DOI:** 10.1186/s13244-019-0722-x

**Published:** 2019-03-29

**Authors:** Massimo Tonolini

**Affiliations:** Department of Radiology, “Luigi Sacco” University Hospital, Via G.B. Grassi 74, 20157 Milan, Italy

**Keywords:** Ileostomy, Colostomy, Contrast enema, Computed tomography (CT), Magnetic resonance imaging (MRI)

## Abstract

Nowadays, large numbers of ileostomies and colostomies are created during surgical management of a variety of intestinal disorders. Depending on indication, surgical technique and emergency versus elective conditions, stomas may be either temporary or permanent. As a result, patients with ileostomies and colostomies are commonly encountered in Radiology departments, particularly during perioperative hospitalisation following stoma creation or before recanalisation, and when needing CT or MRI studies for follow-up of operated tumours or chronic inflammatory bowel diseases. However, the stoma site is commonly overlooked on cross-sectional imaging.

Aiming to improve radiologists’ familiarity with stoma-related issues, this pictorial essay concisely reviews indications and surgical techniques for ileostomies and colostomies, and presents state-of-the art multimodal imaging in patients living with a stoma, including water-soluble contrast stomal enema (WSC-SE), CT and MRI techniques, interpretation and expected findings. Afterwards, the clinical features and imaging appearances of early and late stoma-related complications are illustrated with imaging examples, including diversion colitis.

When interpreting cross-sectional imaging studies, focused attention to the stoma site and awareness of expected appearances and of possible complications are required to avoid missing significant changes requiring clinical attention. Additionally, dedicated imaging techniques such as WSC-SE and combined CT plus WSC-SE may be helpful to provide surgeons the appropriate clinical information required to direct management.

## Key points

- Radiologists often encounter patients with temporary or permanent ileostomies and colostomies
- Particularly in the early postoperative setting, stomas require focused CT/MRI interpretation
- Water-soluble contrast enema may be performed via colostomy or loop ileostomy
- Early stoma-related complications include ischemia/necrosis, retraction and abscess
- Other complications include prolapse, parastomal hernia, obstruction/strangulation and diversion colitis

## Introduction

Intestinal stomas, from the Greek word meaning “mouth”, are temporary or permanent artificial openings of the bowel and are categorised according to the digestive tract segment that is surgically connected to the skin. In the United States, each year approximately 150,000 stomas are created, almost equally divided between ileostomies and colostomies. An estimated 800,000 people currently live with a bowel stoma, of which 40 to 60% will never undergo surgical closure. With an appropriate enterostomal therapy, well-constructed stomas provide patients an acceptable quality of life with few lifestyle limitations. Conversely, complications related to the stoma can have a dramatic impact on patients’ physical and mental health [1].

Aiming to improve radiologists’ familiarity with stoma-related issues, this pictorial essay provides a concise review of indications and surgical techniques of ileostomies and colostomies, then presents state-of-the art multimodal imaging including water-soluble contrast stomal enema (WSC-SE), CT and MRI techniques and expected findings in patients with an intestinal stoma. Afterwards, the key early and late stoma-related complications are discussed with examples, including diversion colitis.

### Indications and surgical techniques

Intestinal stomas are created, either in emergency situations or during elective surgery, to manage a variety of benign and malignant disorders encompassing colorectal cancer (CRC), chronic inflammatory bowel diseases (IBD), acute diverticulitis, bowel perforation, ischemic colitis, radiation colitis, faecal incontinence, intractable perianal sepsis and rectovaginal fistulas. The rationale for stoma creation may be relief of distal obstruction, protection of an anastomosis, temporary bypass or definite diversion of the faecal stream. Depending on the procedure and the indication, stomas can be either permanent or temporary [2–4].

**Fig. 1:**
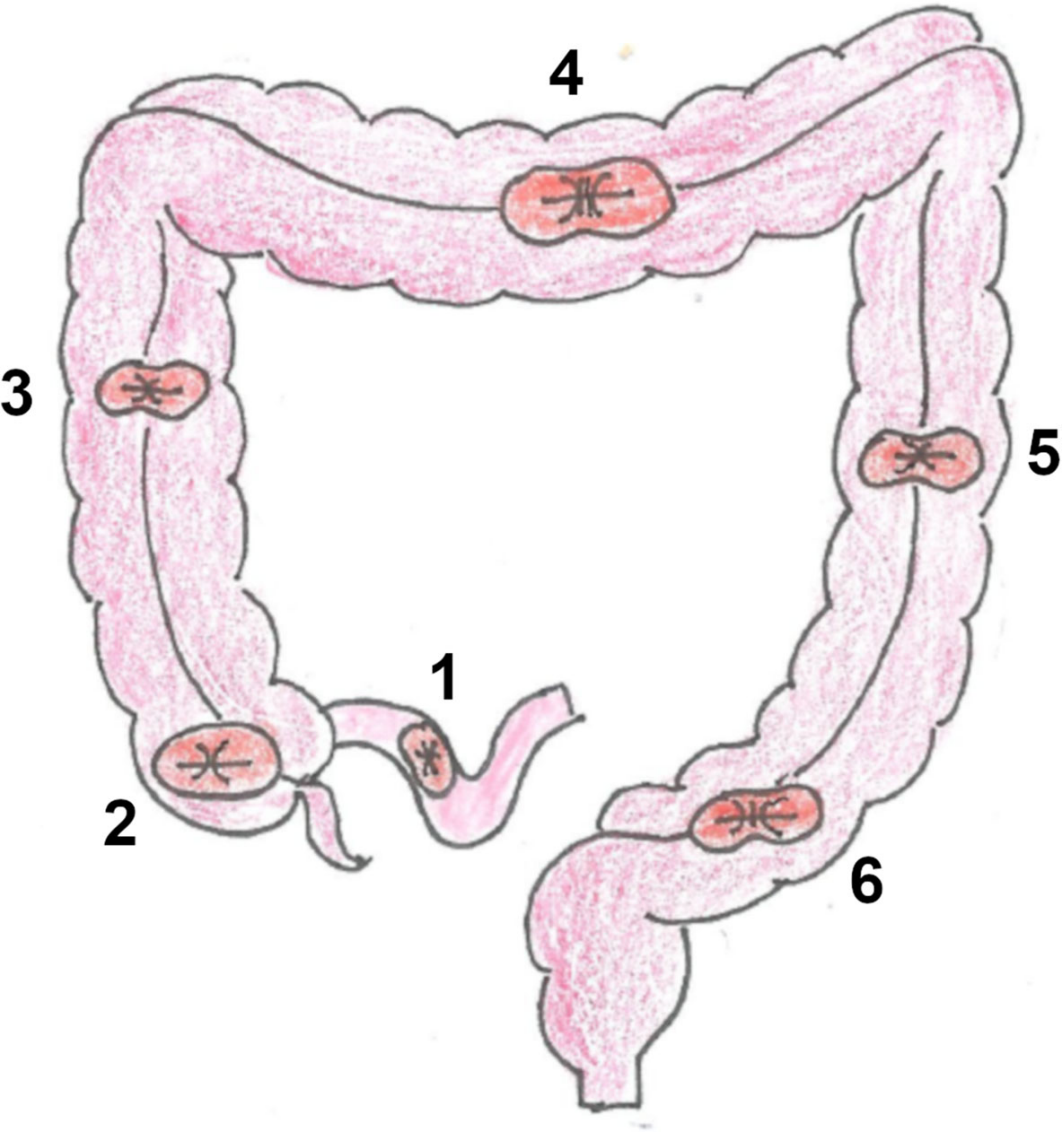
Sites and names of intestinal stomas. Legend: 1) ileostomy, 2) cecostomy, 3) ascending colostomy, 4) transverse colostomy, 5) descending colostomy, 6) sigmoid colostomy

Technically, stomas can be fashioned as either loop or end. In the latter case, the only termination of the bowel opens at the abdominal wall and the other end is either removed or over sewn. An ileostomy involves opening a distal ileal loop through the abdominal wall and is typically located in the right lower quadrant (Fig. 1). The majority of permanent end ileostomies are created after total proctocolectomy for chronic IBD or familial polyposis, when a reservoir (such as an ileal pouch) is not created. On the other hand, colostomies are termed according to the segment of the large bowel that is connected to the skin, and the majority of them are created in the left lower quadrant (Fig. 1). Permanent end colostomies are created when the anorectum is removed such as during abdomino-perineal resection (APR) and when stoma reversal is not possible, such as for unresectable CRC or palliation of incontinence. The most common temporary end colostomy is created during Hartmann’s surgery for acute diverticulitis or CRC of the sigmoid colon, when the closed rectal stump is left in place for future restoration of intestinal continuity [2–4].

Loop stomas are created at bowel segments with a mesentery (such as the ileum, transverse or sigmoid) by pulling out a loop of bowel through the abdominal wall up to its external surface, may be secured using a rod, and are opened on one side to create an afferent (proximal) and an efferent (distal) limb; thus, it has two openings, of which one empties the bowel and the other is connected to the diverted segment. During surgeries with primary bowel-to-bowel anastomosis, a temporary diverting loop stoma is frequently created, in order to improve healing by defunctioning the intestine and minimising the impact of faecal contamination, pressure and tension. These situations mostly include prevention of dehiscence at a high-risk bowel anastomosis, such as after ileocecal resection for Crohn’s disease or during two-stage restorative proctocolectomy with ileal pouch-anal anastomosis for ulcerative colitis. After a variable delay (two to six months), temporary ileostomies are generally reversed (closed) to re-establish the intestinal continuity. Alternatively a double-barrelled colostomy may be created, which is similar to loop stomas because it has two openings, when after resection of a diseased tract both limbs are exteriorised at the same site at the abdominal wall [2–4].

### Water-soluble contrast stomal enema

#### Rationale and indications

Surgeons commonly request WSC-SE via colostomy or loop ileostomy to either A) check for integrity of a distal bowel anastomosis, particularly in patients at high risk of complications, or B) assess the bowel anatomy, residual length and possible stricture before recanalisation. For instance, at our Institution WSC-SE is generally performed before ileostomy takedown after restorative proctocolectomy and before recanalisation of Hartmann’s surgery. However, routine WSC-SE prior to closure of defunctioning ileostomy remains controversial, since some studies shown that it rarely provides additional information leading to modification of patient management [5]. The key contraindication for WSC-SE is radiographically confirmed bowel obstruction, a situation in which the use of CT is advisable and which will be discussed later on.

#### Technique

**Fig. 2:**
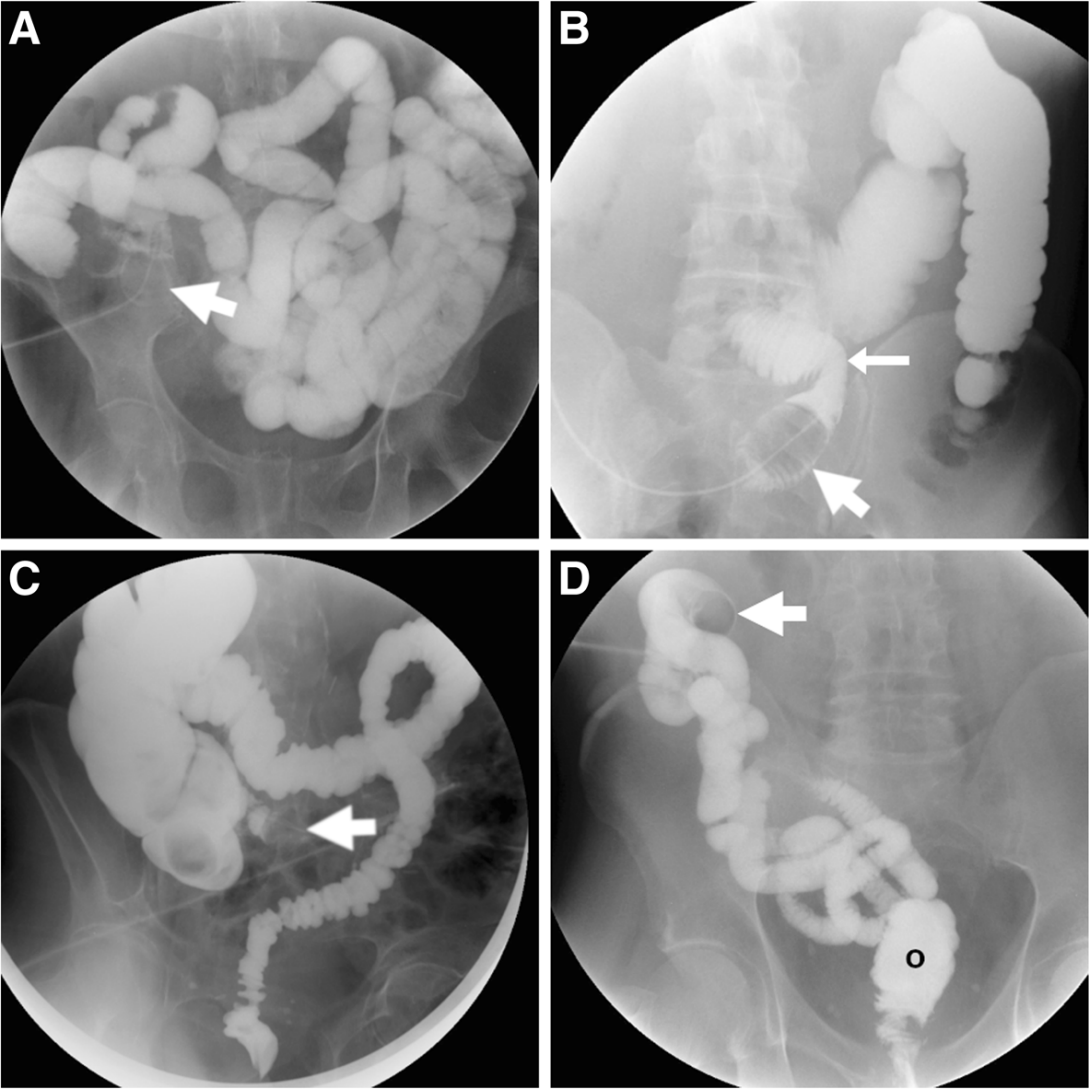
Technique and normal appearances of water-soluble contrast enema (WSC-SE) using an inflated Foley catheter (thick arrows) passed through the stoma. **a** In a patient with long-standing Crohn’s disease, WSC-SE performed via end ileostomy in the right iliac fossa shows the radio-opaque diluted contrast medium filling the upstream small bowel, without signs of stricture or dilatation. **b** Following right colectomy, WSC-SE at defunctioning ileostomy depicts the normal termino-lateral ileo-transverse anastomosis (arrow) without anastomotic leakage. **c** After recent sigmoidectomy with primary anastomosis, WSC-SE shows patent residual colon up to the rectum, without extraluminal leakage. **d** Before planned ileostomy takedown, after restorative proctocolectomy for ulcerative colitis, WSC-SE via the efferent limb opacifies the small bowel up to the newly created ileal pouch (o)

**Fig. 3:**
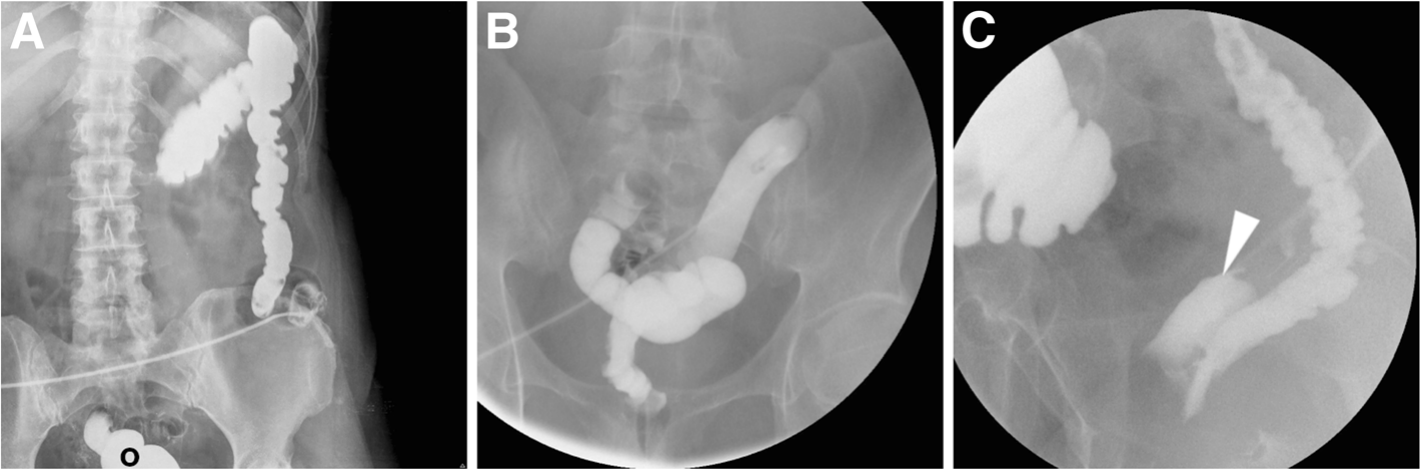
Three examples of WSC-SE performed via colostomy. **a** After recent Hartmann’s surgery, retrograde opacification of closed rectal stump (o) was performed prior to WSC-SE at the descending colostomy. **b** In a different patient, WSC-SE performed at the efferent limb of a double-barrelled colostomy at the left iliac fossa depicts the diverted rectosigmoid tract. **c** at the end of a WSC-SE study, contrast opacifies the vagina (arrowhead) indicating the presence of a rectovaginal fistula

Apart from fasting, patients with an ileostomy do not require any special preparation. Conversely, prior to WSC-SE colostomies should receive preliminary bowel cleansing using irrigation, using the same manoeuvers many patients already use to regulate their stoma. The patient is placed supine on the fluoroscopy table. After removing the stoma appliance and cleaning the peristomal skin, the radiologist or the referring surgeon may perform a gentle examination of the stoma using the little finger, in particular to identify the two openings of a loop or double-barrelled stoma. A lubricated urinary-type Foley catheter is introduced into the stoma approximately 15 cm into the bowel lumen and inflated using a few milliliters of saline. Then, the catheter is connected to the nozzle of the enema bag, filled with water-soluble contrast medium such as 1:1 diluted diatrizoate meglumine - diatrizoate sodium solution (Gastrografin®, Bracco, Milan, Italy). The attending radiologist witnesses the progressive filling of the bowel lumen connected to the stoma on fluoroscopy, and captures appropriate panoramic and focused frontal, oblique and panoramic views (Fig. 2). Examples of WSC-SE performed at ileostomies and colostomies are shown in Figs. 2 and 3, respectively.

**Fig. 4:**
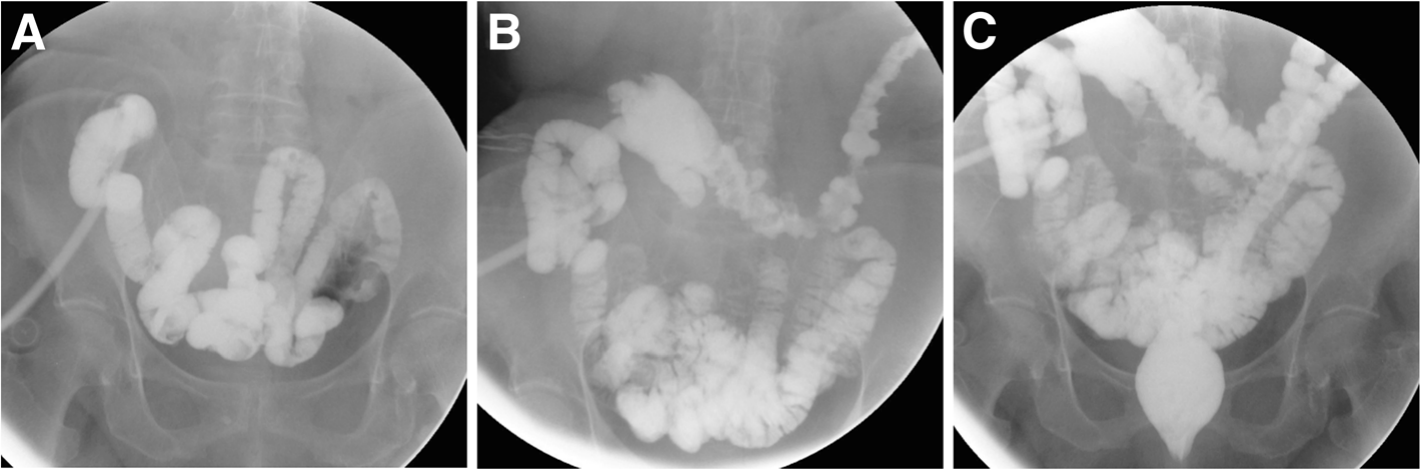
Double WSC-SE at a loop ileostomy following recent ileocecal resection for Crohn’s disease, performed via the afferent (**a**) and efferent (**b**, **c**) limbs, obtaining opacification of the residual large bowel up to the rectum

The most common abnormal findings include extraluminal contrast leakage at the anastomosis and opacification of a fistula (Fig. 3C). At the surgeon’s request, loop and double-barrelled stomas may receive WSC-SE along both afferent (upwards) and efferent (downwards) limbs (Fig. 4). Furthermore, when combined WSC-SE and rectal enema is required (such as before colostomy takedown following Hartmann’s surgery, Fig. 3A), the latter should be performed initially since contrast in the right colon and distal ileum can mask the closed distal stump.

### Cross-sectional imaging of intestinal stomas

#### CT and CT-enterography: Technique and interpretation

Nowadays, the vast majority of operate patients with an intestinal stoma commonly receive contrast-enhanced CT, mostly to investigate suspected postoperative complications or during oncologic follow-up. Additionally, patients with Crohn’s disease are frequently investigated using CT-enterography after gradual ingestion of iso-osmotic material such as polyethylenglycole (PEG). Before CT-enterography, patients with an ileostomy should not receive laxatives but liquid diet only. Due to the short transit time through the bowel to the stoma (usually about 20 min), to obtain the best diagnostic result patients should be placed on the scanner table immediately when the ingested solution begins to fill to stoma bag [6].

**Fig. 5:**
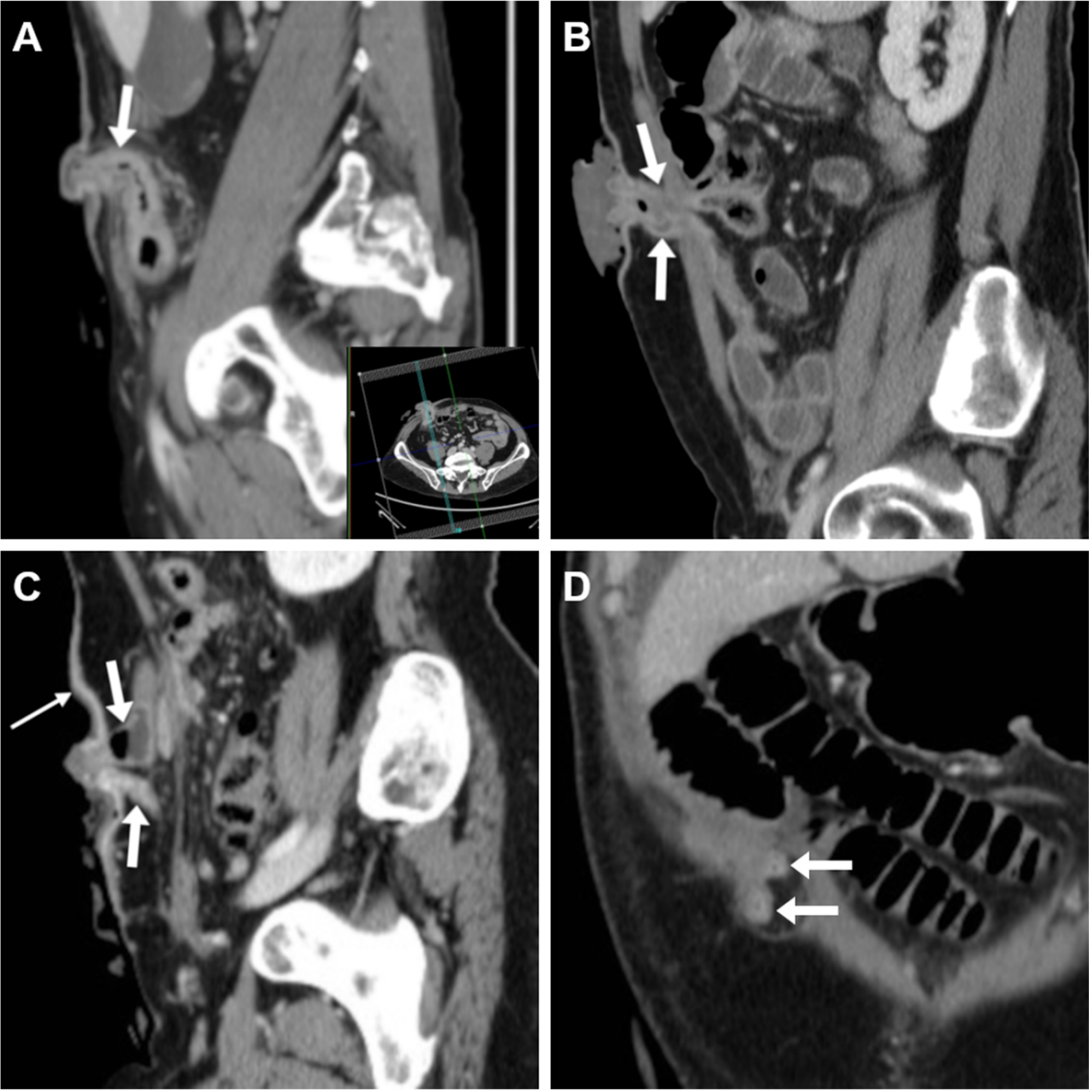
CT reconstruction technique and expected findings of ileostomies. **a** Focused oblique-sagittal (note lateral obliquity in inset) image of a normal end ileostomy with uniform mural thickness and enhancement of the stomal tract (arrow), mild protrusion at external orifice. **b** oblique-sagittal images of a loop ileostomy identify non-thickened, collapsed efferent and afferent limbs (arrows). Note fluid flowing at the external orifice indicating stomal patency. **c**, **d** A typical defunctioning loop ileostomy with two limbs (arrows) on sagittal (**c**) viewing and coronal (**d**) appearance. Note mild thickening and hyperenhancement of peristomal skin in **c** (compared to **a** and **b**) consistent with cutaneous inflammation at physical examination

**Fig. 6:**
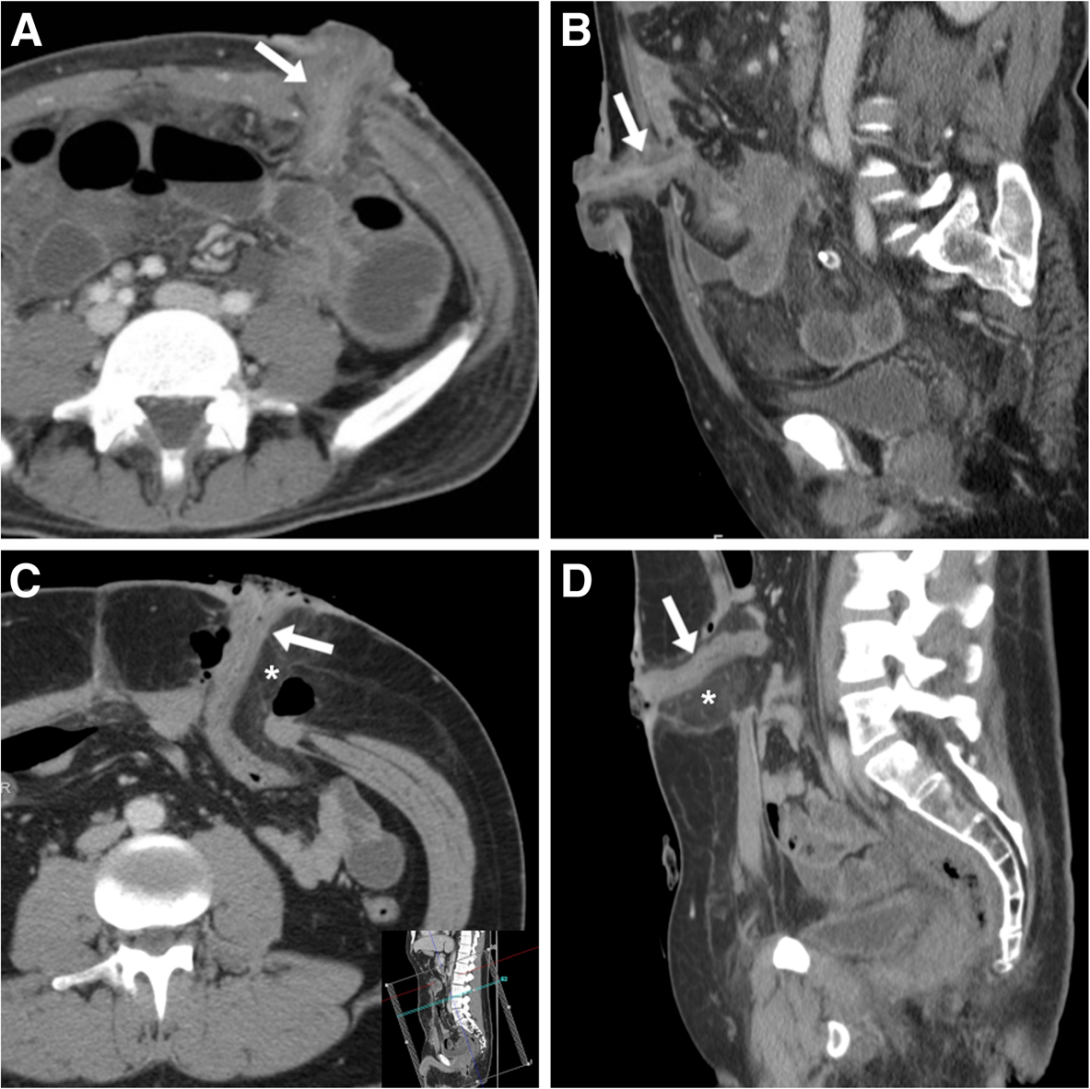
Expected early CT findings in newly created stomas. **a**, **b** A typical recent ileostomy, with collapsed stomal tract (arrows) showing mild uniform mural stratification (enhancing mucosa and oedematous submucosa). **c**, **d** Uncomplicated descending colostomy created 4 days earlier during low anterior rectal resection, with stomal tract (arrows) showing similar features to **a**, **b**, surrounded by oedematous fat stranding (*) and gas bubbles as expected postsurgical findings

However, the stoma site is commonly overlooked by radiologists interpreting CT studies. Particularly during the early postsurgical hospitalisation after stoma creation, careful scrutiny of ileostomies and colostomies is warranted to avoid missing significant abnormalities. Focused oblique-sagittal CT images parallel to the stomal segment should be reconstructed, along a plane that is usually tilted ventrally 10° to 30° to the ipsilateral side of the abdomen (Fig. 5A, see inset) and slightly downwards (Fig. 6C, see inset).

#### Expected CT findings

The usual CT appearance of an intestinal stoma is a collapsed colonic or ileal loop with uniform mural thickness and homogeneous contrast enhancement that traverses the fascial planes of the anterior abdominal wall, reaches the external opening and protrudes approximately 1–1.5 cm at the cutaneous surface (Fig. 5A). Enteral or fecal material flowing through respectively ileostomy or colostomy into the stomal bag indicates patency of the stomal tract (Fig. 5B). When assessing loop and double-barrelled stomas, both the afferent and efferent limbs should be identified (Fig. 5B– D). During the early postoperative days, mild uniform thickening of the stomal tract due to oedematous submucosa, inflammatory-type peristomal fat stranding and gas bubbles are commonly observed and should not be considered abnormal findings (Fig. 6). Additionally, identification of thickened and hyperenhancing peristomal skin (Fig. 5C) should be reported as suggestive of inflammation and requiring clinical attention [7, 8].

#### Combined CT with stomal enema

**Fig. 7:**
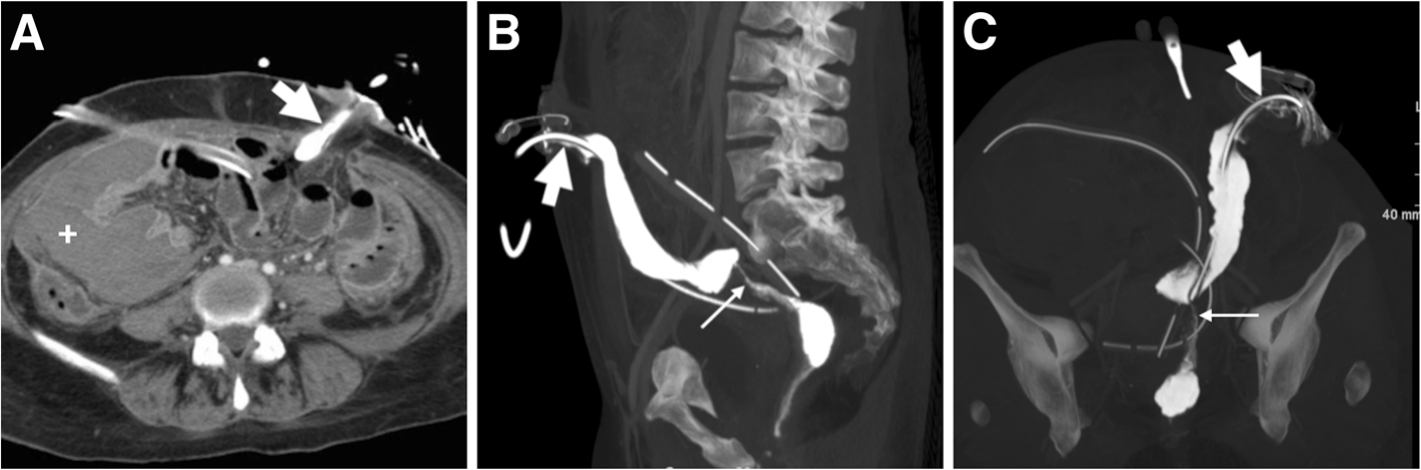
Combined CT plus WSC-SE in a patient with iatrogenic rectal perforation during stricture dilatation. Axial CT image (**a**) showing large hyperattenuating collection in the right abdomen (+) and diluted iodinated contrast medium injected in the cannulated efferent limb of the loop colostomy (thick arrows). Maximum-intensity projection reconstructions (**b**, **c**) depict the contrast medium-filled diverted rectosigmoid colon with stricture (thin arrows), without extraluminal leakage. Note drainage tubes in place

In selected patients, performing a one-stop-shop CT plus WSC-SE may be beneficial to provide combined anatomic and functional/dynamic information. This technique includes a preliminary unenhanced acquisition of the abdomen followed by a contrast-enhanced CT scanning after cannulation of the stoma and injection of iodinated contrast medium such as 5–10% diluted iopamidol (Bracco, Milan, Italy) or iopromide (Schering Pharma, Berlin, Germany) (Fig. 7).

#### MRI and MR-enterography

Particularly in the setting of IBD, patients with intestinal stomas increasingly receive MRI studies. Both CT- and MR-enterography are currently recommended by the European Crohn’s and Colitis Organization (ECCO) guidelines as then current standards for evaluation of the small bowel, and the latter is preferred in young patients who need several imaging studies during their lifelong disease [9].

**Fig. 8:**
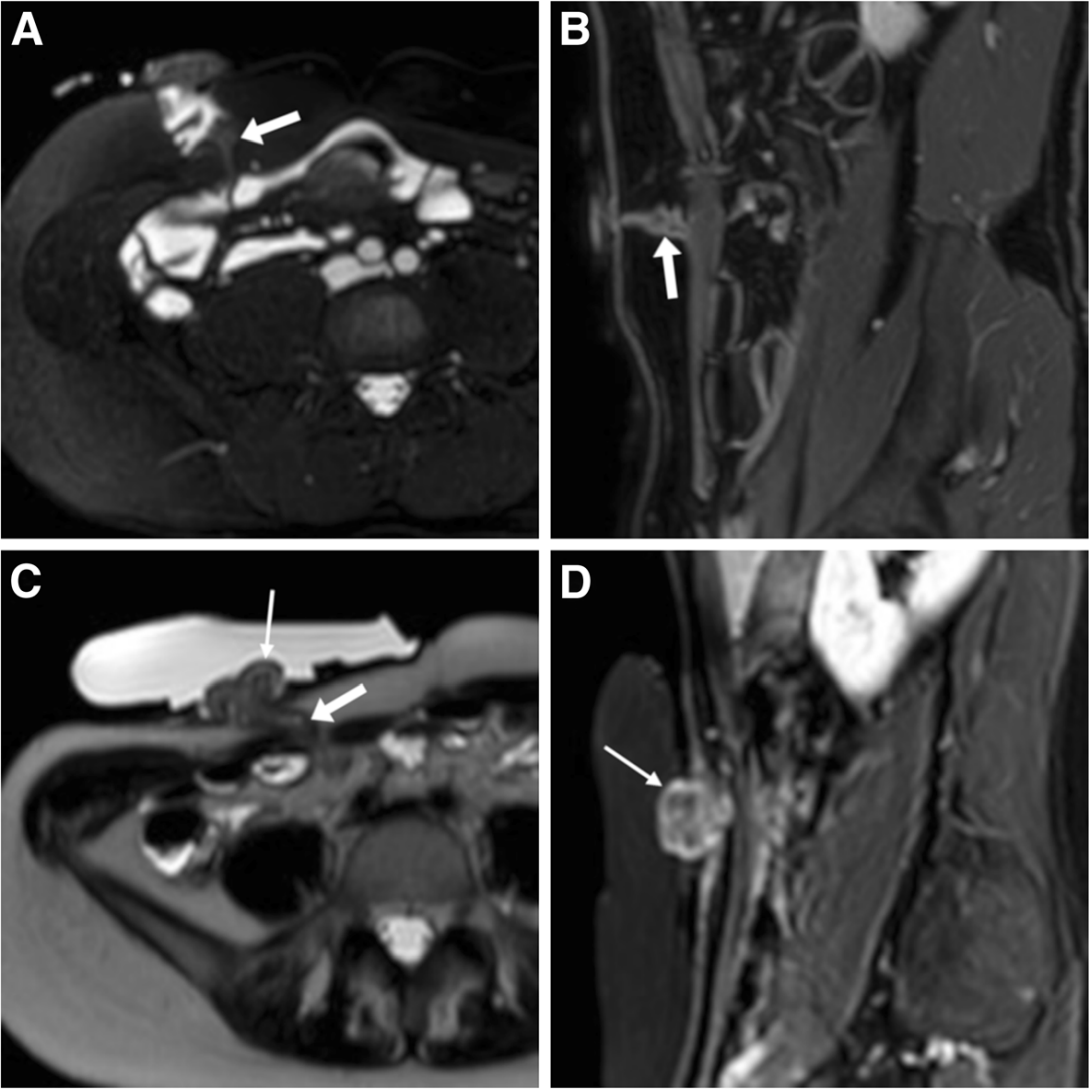
Normal appearance of ileostomies at MR-enterography in two patients operated for Crohn’s disease. **a**, **b** Axial fat-suppressed T2-weighted (**a**) and sagittal post-gadolinium fat-suppressed T1-weighted (**b**) images showing collapsed stomal tract (arrow) traversing the abdominal wall, with normal mural thickness and contrast enhancement comparable to that of other small bowel loops. **c**, **d** Axial T2- (**c**) and post-gadolinium fat-suppressed T1-weighted (**d**) images showing collapsed stomal tract (arrow), mild protrusion at external orifice (thin arrows), and abundant fluid flowing within stoma bag consistent with stomal patency

Although discussion of MRI protocols lies beyond the scope of this article, MR-enterography relies on ingestion of iso-osmotic biphasic material such as PEG solution. Such as during CT-enterography, also in the MRI suite patients with an ileostomy should be scanned just when the ingested solution reaches the stoma. MRI and MR-enterography well depict the normal, collapsed stomal tracts crossing the abdominal wall, with uniform mural thickness and contrast enhancement comparable to that of other small bowel loops, mild protrusion of the external orifice (Fig. 8A, B). At MR-enterography, fluid flowing from the stoma into the bag confirms bowel and stomal patency (Fig. 8C, D) and peristomal skin inflammatory changes are readily identified (Fig. 12) [8, 10].

### Stoma-related complications: An overview

In the literature, reported rates of stoma-related morbidity vary from 2.9% to 81.1% according to stoma type, indication and underlying disease, elective or emergent surgery, patient factors (such as obesity and comorbidities) and definition of complications. The incidence of complications is higher in end colostomies, followed by loop colostomies and ileostomies. Conversely, small bowel obstruction more commonly occurs with the latter [11].

Stoma-related complications may occur early or late after creation. The very common peristomal skin and metabolic (dehydration, electrolyte imbalance) problems will not be discussed in this radiological review. Within 30 days from surgery, common early complications include ischemia/necrosis, stoma retraction and parastomal abscess. Conversely, late occurrences include parastomal hernia (PH), stomal prolapse and varices. Bowel obstruction and strangulation may occur both as early or late complications [12, 13].

### Early stomal complications

#### Stomal ischemia and necrosis

Ischemia results from inadequate blood supply to the recently created stomal tract and most usually complicates colostomies, in association with obesity and emergency operations. Although rare, necrosis is generally a severe event which may require emergency surgical revision, depending upon the depth of the ischemic segment. Vascular impairment to the newly created stoma may be either superficial (in 2–13% of patients) or deep to the fascia (0.37–3%). Necrosis is diagnosed clinically at physical examination, and emergency CT may be performed to assess the depth of the hypo- or nonenhancing ischaemic bowel conduit [12, 13].

#### Stomal retraction

**Fig. 9:**
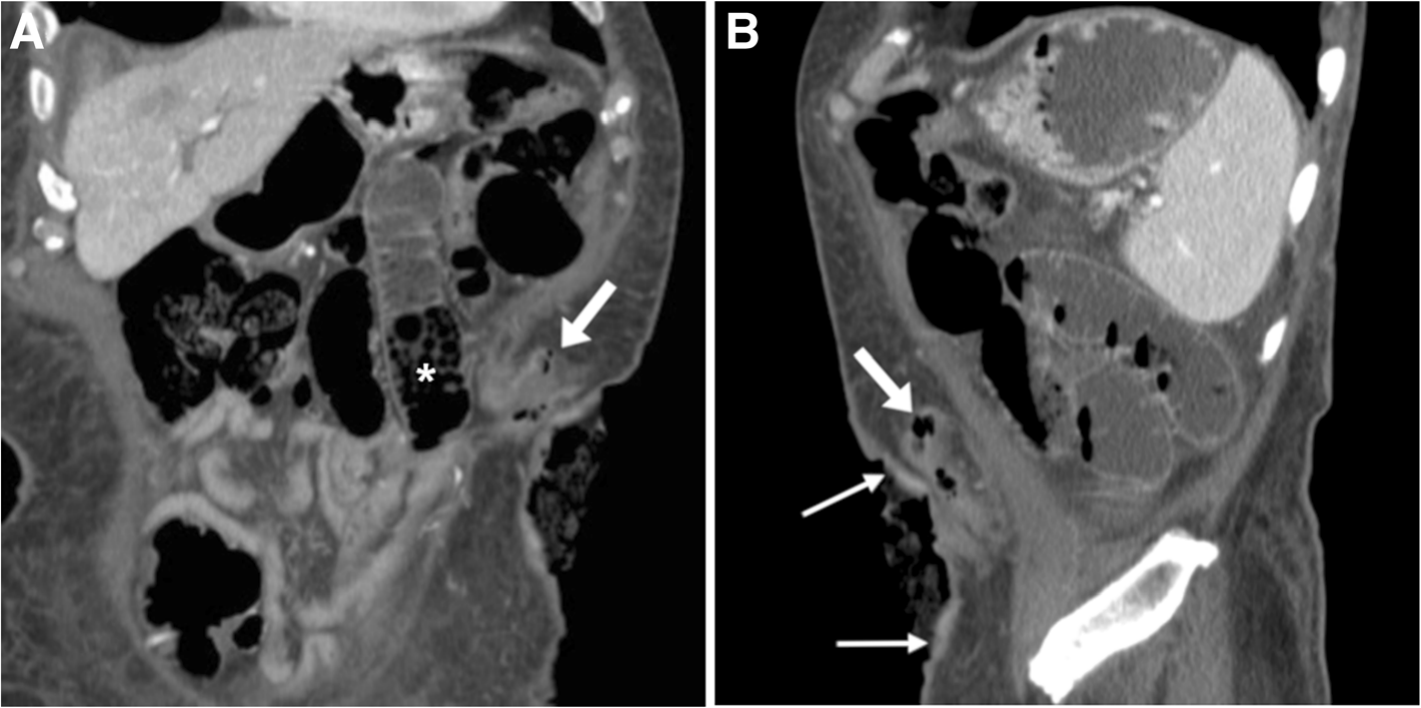
Stoma retraction appearing as depressed, thickened and irregular skin (thin arrows) at the site of a descending colostomy with elongated collapsed tract (arrows). Note distended small bowel loops with feces (*) consistent with obstruction

Retraction occurs in 1.4–9% of patients with both ileo- and colostomies. During the early postoperative period, retraction is caused by excessive tension on the connected bowel loop, and if untreated may cause long-term stricture. Later, retraction may develop with or without a coexisting PH, and should be suggested when imaging shows loss of the normal external bulging or cutaneous depression (Fig. 9).

#### Stomal abscess

**Fig. 10:**
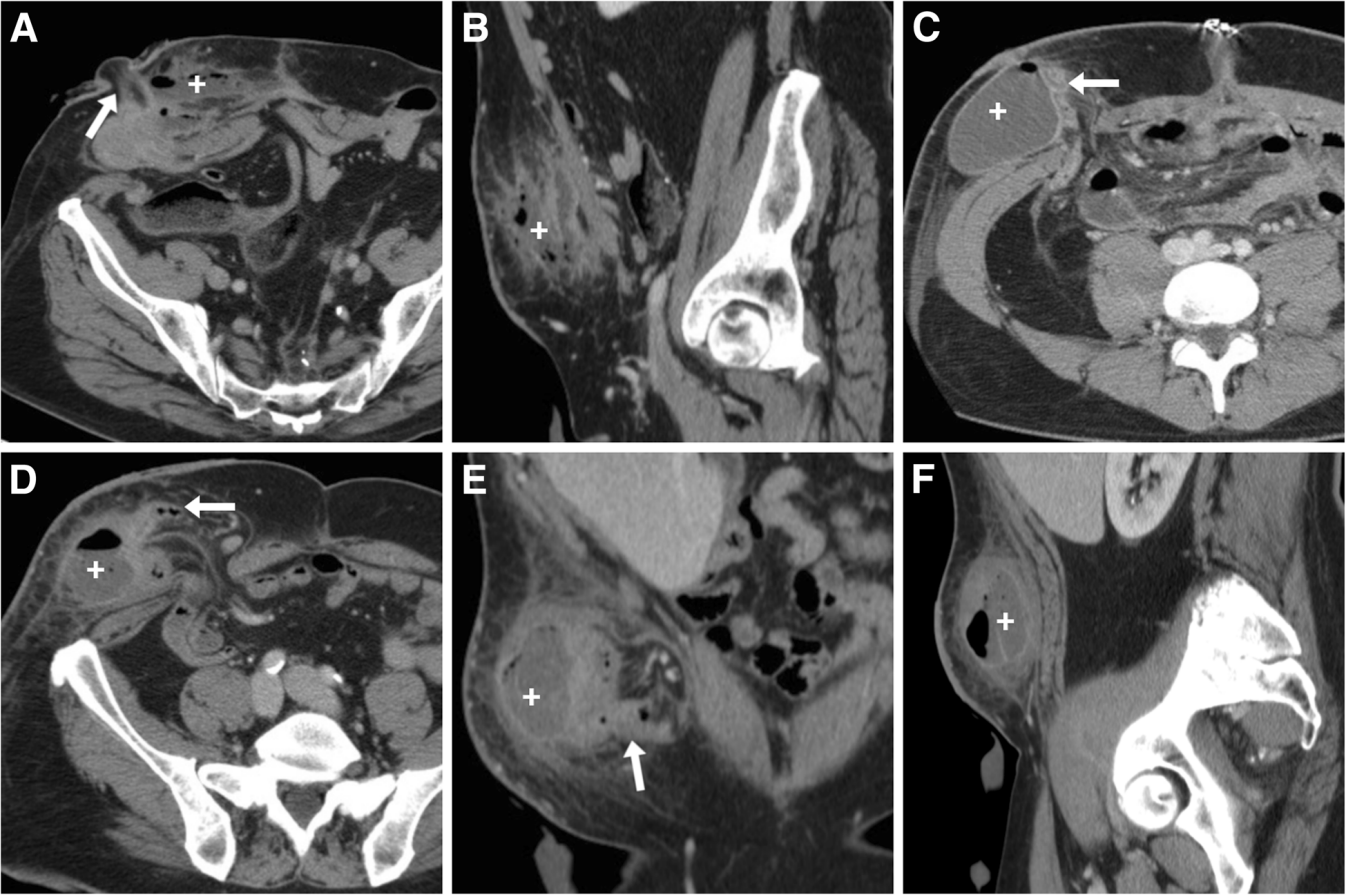
Three cases of stomal abscesses, all of which required surgical treatment. **a**, **b** Ill-defined collection (+) with mixed fluid and air content occupying the subcutaneous fat at the site of a recent ileostomy (arrow in **a**). **c** Large fluid collection (+) with thin walls abutting the lateral aspect of the ileostomy (arrow), following recent anterior rectal resection. **d**-**f** A typical abscess (+) with thickened enhancing walls and air-fluid level within a parastomal hernia (PH), following colectomy for Crohn’s disease

Most usually developing within the first weeks after surgery, at cross-sectional imaging abscesses show the well-known appearance as mixed fluid and gaseous collections of variable size with enhancing periphery, located in the subcutaneous tissue abutting the stomal tract, usually surrounded by inflammatory fat stranding (Fig. 10) [3, 12, 13].

### Long-term stomal complications

#### Stomal prolapse

**Fig. 11:**
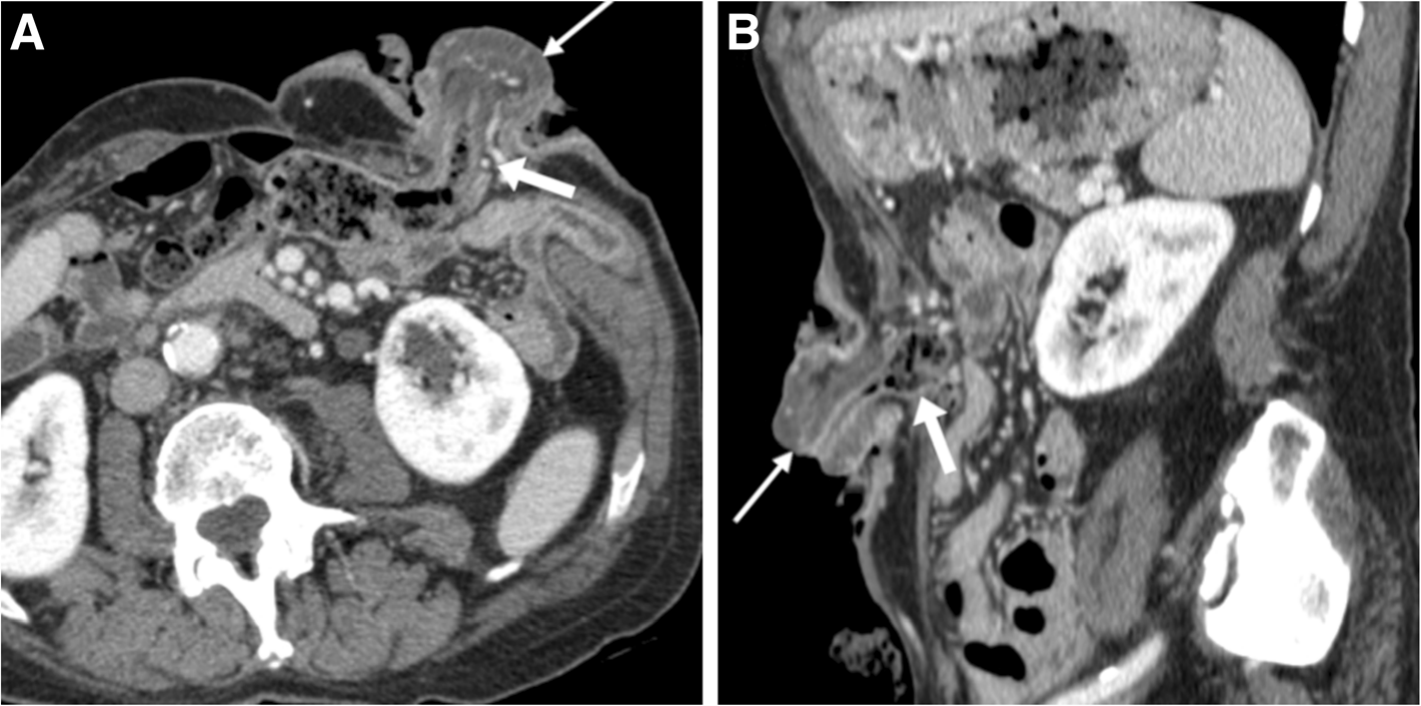
Clinically confirmed protrusion of an ileostomy with markedly oedematous stomal tract (arrows) and bulging of external orifice (thin arrows), which was managed conservatively

Prolapse refers to excessive, full-thickness protrusion of the stomal tract bowel at the cutaneous opening and occurs in approximately 3% of ileostomies and 2% of colostomies, in association with risk factors such as advanced age, obesity and obstruction at time of creation. Sometimes associated with a PH, prolapse may either fixed or sliding (intermittent, related to increased abdominal pressure and reducible). Prolapse may be identified at cross-sectional imaging (Figs. 8C, 11), tends to involve the efferent limb of loop stomas, and may require surgical revision, resection or relocation [2].

#### Stomal recurrence of Crohn’s disease

**Fig. 12:**
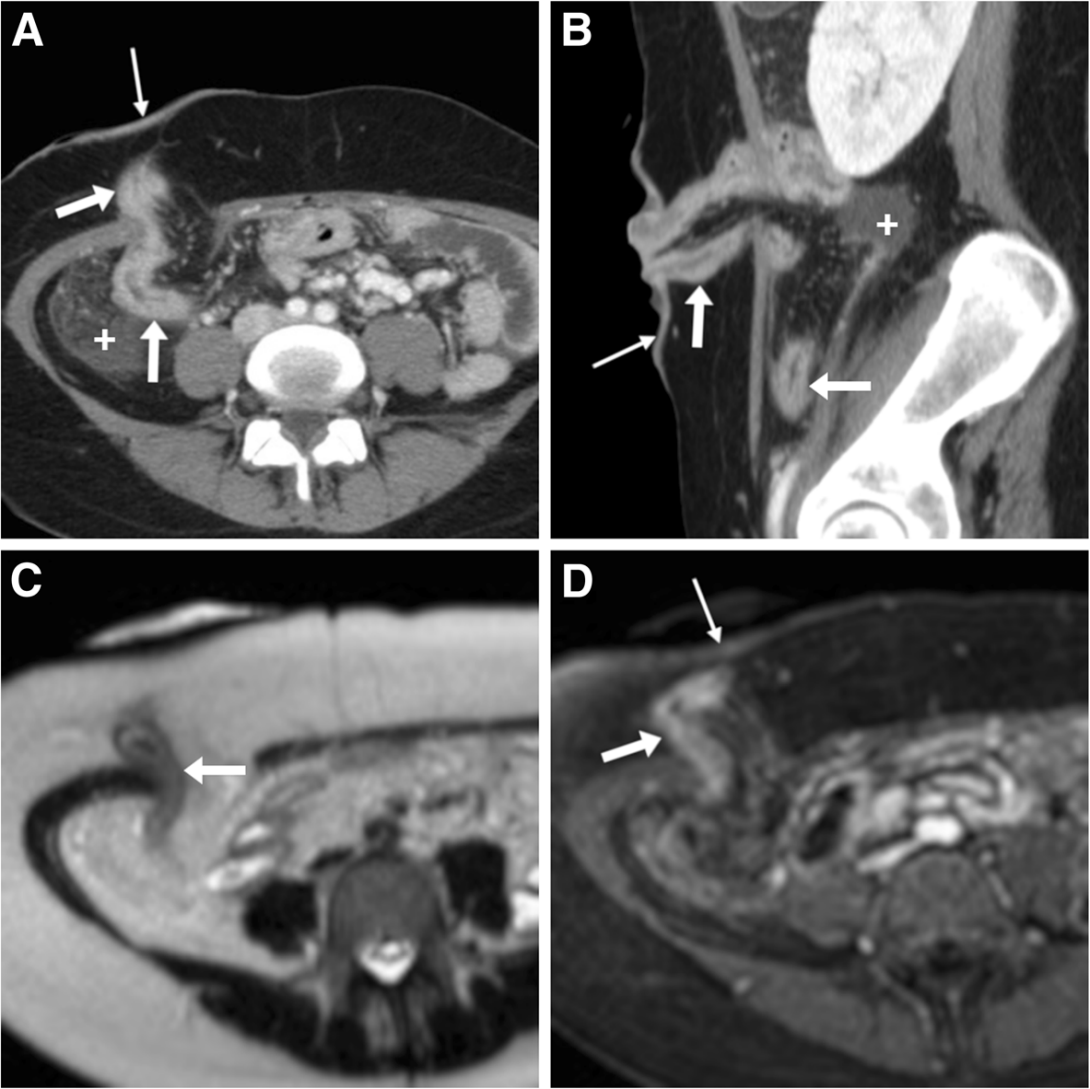
Reactivation of Crohn’s disease at an ileostomy. **a**, **b** CT showing diffuse, mildly irregular mural thickening with marked contrast enhancement along the stomal tract and the distal small bowel, consistent with active disease (also note fat stranding and fluid in the right parietocolic gutter). **c**, **d** In the same patient, MR-enterography confirms thickened walls on T2-weighted (**c**) with hyperenhancement on post-gadolinium fat-suppressed (**d**) images. Additionally, note enhancing inflamed peristomal skin (thin arrows in **a**, **b** and **d**)

A not-unusual diagnosis at CT- and MR-enterography in operated patients, recurrent Crohn’s disease at an ileostomy shows the usual cross-sectional features of the treated IBD along the stomal tract, such as circumferential mural thickening with intense or stratified enhancement (Fig. 12) [8, 14].

#### Parastomal hernia

A subtype of incisional hernia, PH is defined by the European Hernia Society (EHS) as an abnormal protrusion of contents of the abdominal cavity through the abdominal wall defect created during placement of a colostomy or ileostomy [15].

Although difficult to assess due to lack of a uniform definition and inadequacy of physical examination, the prevalence of PH progressively increases over time. Within 5 years, some degree of PH is reported in 48% of end colostomies and 28.3% of end ileostomies. The incidence is much lower in loop ileostomies (6.2%) since they are generally temporary stomas and PH has not sufficient time to develop. Risk factors include advanced age, obesity, cancer, diabetes, malnutrition, increased intra-abdominal pressure and chronic obstructive lung disease. Nowadays, primary prevention with prophylactic mesh placement during stoma creation is the established measure to decrease the rate of PH development. The majority (75%) of patients complain from PH-related symptoms such as peristomal bulging during cough, pain, discomfort, difficulty holding the stoma appliance in place, leakage of bowel contents and peristomal dermatitis. Indications for surgical repair include obstruction, incarceration, prolapse, stenosis and malfunctioning, large size and patient preference due to pain, intractable dermatitis or cosmetic reasons [13, 16, 17].

Nowadays, CT is recognised as the technique of choice to 1) detect smaller, clinically non-apparent PH, 2) confirm the clinical diagnosis and 3) characterise content and stage the PH. Aiming to decrease uncertainty among different surgical classifications, the EHS categorises PH according to the size of the abdominal wall defect and on the coexistence of another incisional hernia (Table 1) [15].

**Table 1:** Clinical classification of parastomal hernias by the European Hernia Society (EHS). Additionally, parastomal hernias are categorised as either primary (P) or recurrent (R)

	Size of the abdominal wall defect	
	Small (< 5 cm)	Large (> 5 cm)
Concomitant incisional hernia	I	III
Absent incisional hernia	II	IV

**Fig. 13:**
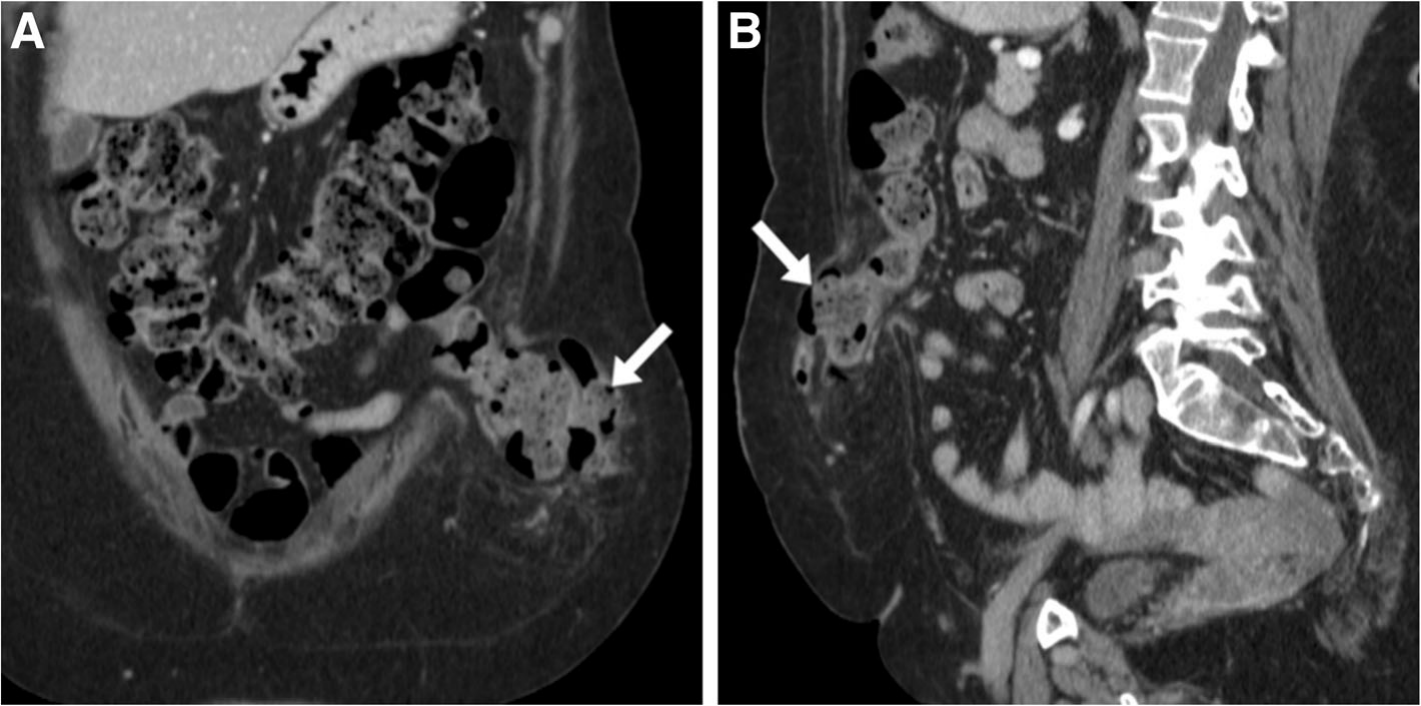
Elongated, patent permanent descending colostomy (arrows) containing feces in a patient with history of abdomino-perineal resection, without formation of hernia sac, consistent with type 0 PH

**Fig. 14:**
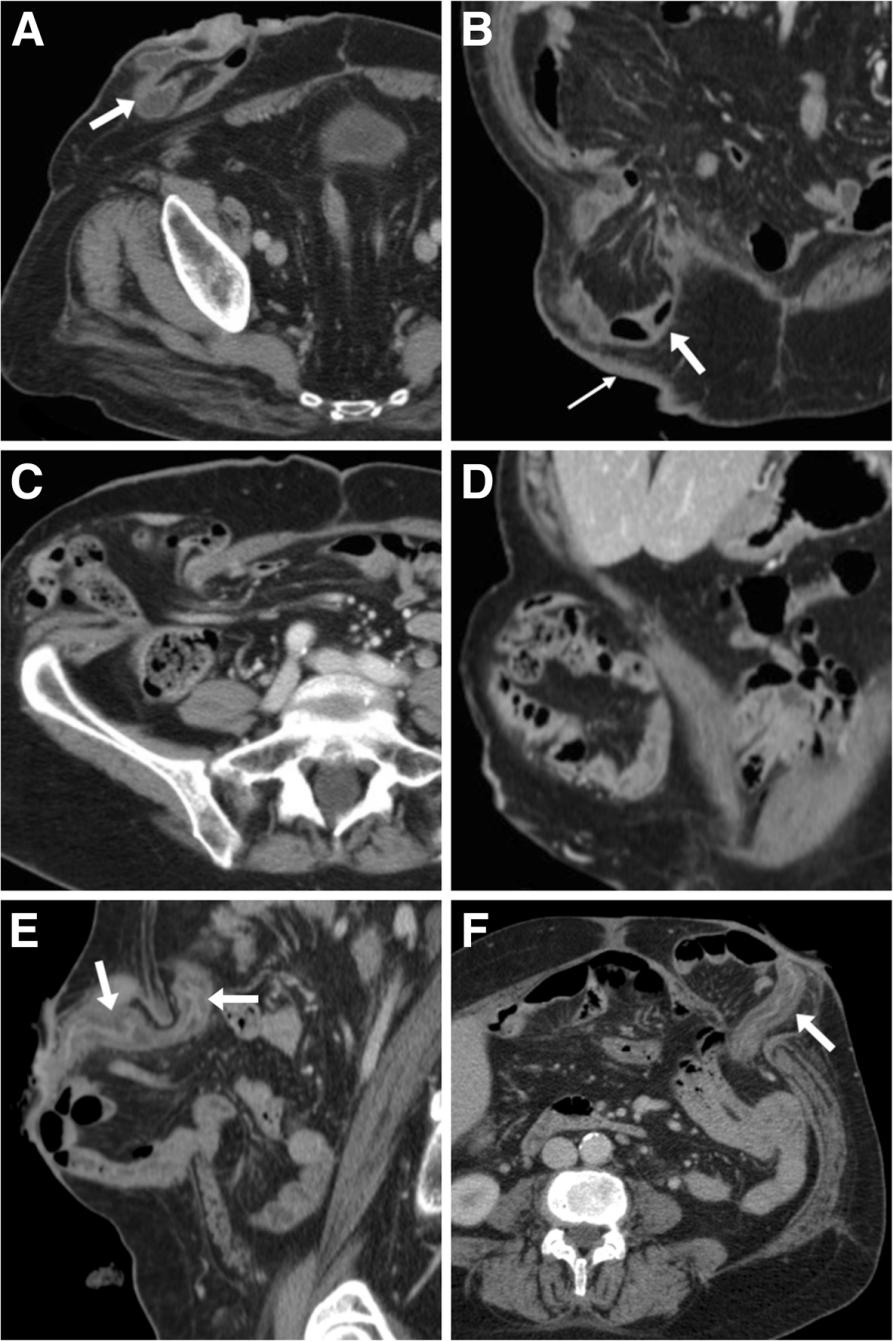
Three cases of uncomplicated PH. **a**, **b** CT type Ib PH measuring approximately 6 cm in size containing a redundant ileostomy tract (arrows). **c**, **d** Larger PH containing a large portion of the right colon. **e**, **f** Type III PH following recent Hartmann’s surgery containing the oedematous colostomy tract (arrows) and some small bowel loops

Additionally, a CT-based staging system (Table 2) has been proposed, which relies on the size and content of the hernia sac. A long, redundant stomal tract in long-standing PH (grade 0, Fig. 13) is considered a normal appearance. The key differentiation provided by CT is between grade I and grade III hernias, the latter containing other bowel loops than the stomal tract (Fig. 14) [18, 19].

**Table 2:** CT-based classification system of parastomal hernias. Note: size of the hernia sac differs from that of the abdominal wall defect in EHS clinical classification (Table 1)

CT Type	Description
0	Peritoneum follows the wall of the bowel forming the stoma, without sac formation
Ia	Hernia containing bowel loop forming the colostomy with sac < 5 cm
Ib	Hernia containing bowel loop forming the colostomy with sac < 5 cm
II	Hernia containing omentum
III	Hernia containing other intestinal loop than the bowel forming the stoma

**Fig. 15:**
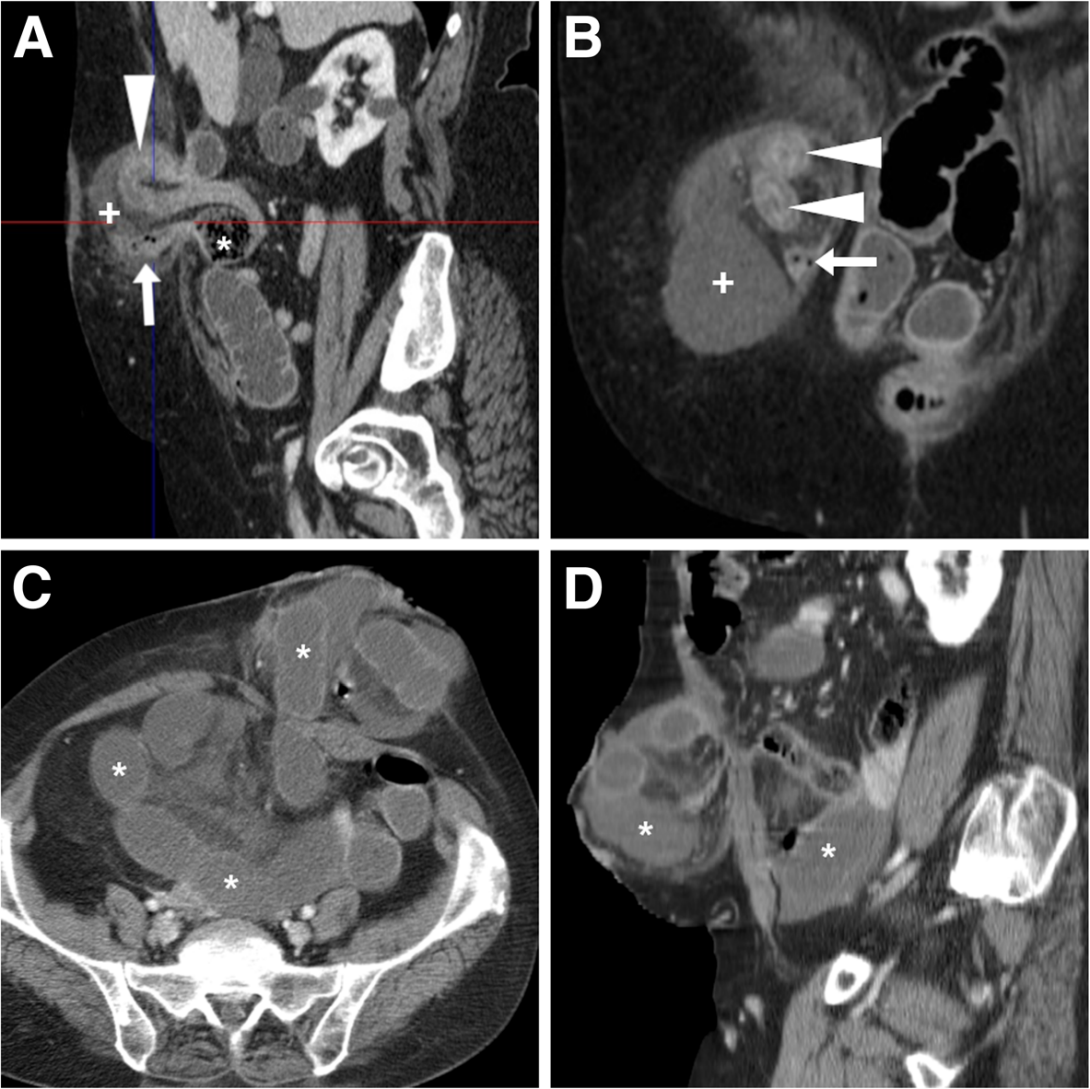
Two surgically treated complications of PH. **a**, **b** Right-sided PH containing ileostomy tract (arrows) and another herniated small bowel (arrowheads) in a closed-loop obstructive pattern with markedly thickened walls and hypoenhancing submucosa, consistent with ischemia. **c**, **d** Strangulation with extensive small bowel infarction in a patient with PH at permanent colostomy: note dilated fluid-filled enteric loops (*), some of them with non-enhancing walls indicating necrosis

Furthermore, the presence of a PH is commonly involved in the development of further complications such as stomal retraction, abscess (Fig. 10), prolapse, closed-loop obstruction and strangulation (Fig. 15) [13, 16, 17].

### Stoma-related bowel obstruction and strangulation

**Fig. 16:**
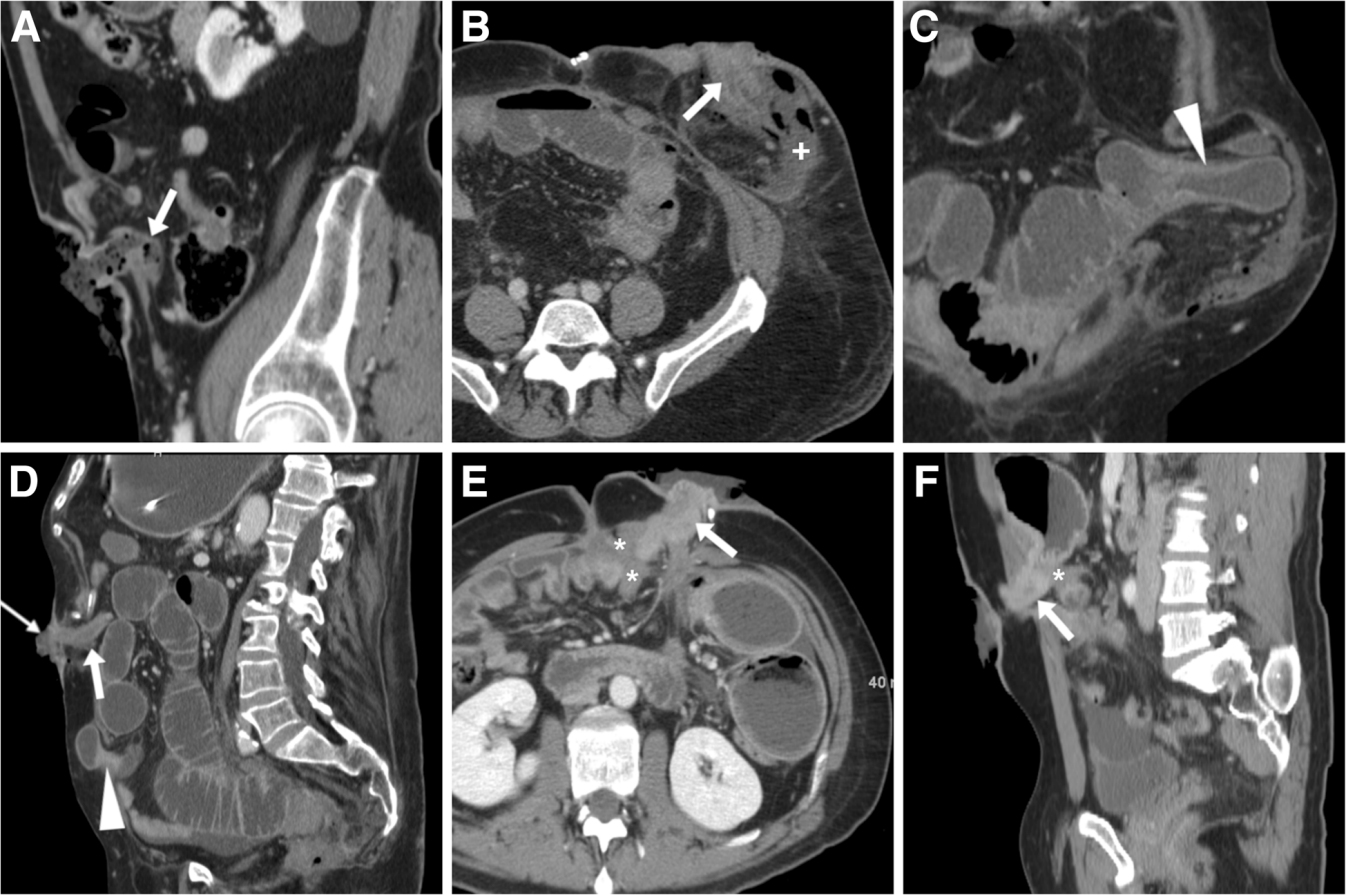
Surgically confirmed obstructed stomas and alternative diagnoses. **a** Sagittal image showing sharply angulated stomal tract (arrow) causing upstream obstruction. **b**, **c** Entrapment with upstream dilatation of large bowel (arrowhead in **c**) within a large PH in a patient with Crohn’s disease and permanent ileostomy (arrow in **b**). Note peristomal fluid (+) and gas bubbles consistent with recent surgery. **d** Incisional hernia (arrowhead) causing obstruction, in a patient with uncomplicated ileostomy (arrow). **e**, **f** Blockage of ileostomy (arrow) from solid tissue (*) consistent with peritoneal carcinomatosis

Mechanical obstruction occurs in at least 5% of patients with a stoma and may result from a variety of causes, related to the stoma, previous surgery or underlying disease. In the vast majority of situations, CT represents the ideal technique for investigating patients with clinical or plain radiographic signs of obstruction. In patients with ileostomies and colostomies, CT generally allows identification of the transition point and is therefore useful to differentiate local (stoma-related) causes such as a twisted stomal tract (Fig. 16A) or entrapment within a PH (Fig. 16B,C), from more proximal obstruction causes such as intra-abdominal adhesions or other hernia (Fig. 16D) and alternative diagnoses such as bowel encasement of loops by neoplastic tissue (Fig. 16E,F) [3, 8].

Closed-loop obstruction refers to obstruction with two adjacent transition points and may be further complicated by strangulation. Findings consistent with ischaemia include C-shaped loop, thickened walls, absent or diminished mural enhancement, mesenteric fluid (Fig. 15) [20].

### Diversion colitis

Initially described in 1981, diversion colitis (DC) refers to a nonspecific mucosal inflammation of diverted rectal or colonic segments by a loop or end colostomy. The mechanism is poorly understood and may involve stasis, ischaemia, loss of essential nutrients from enteric bacteria or overgrowth of harmful bacteria in the diverted segment. The unspecific endoscopic appearances include mucosal erythema, oedema, friability, granularity and erosions. Corresponding histologic patterns include mild or moderate chronic and acute inflammatory changes, crypt atrophy and distortion, follicular lymphoid hyperplasia. Within a few months from initial surgery, DC tends to develop in nearly all patients with diversion created for any pathology. Features consistent with DC are reported in 91% of patients with pre-existing IBD and 70-74% of other patients, without association with sex, age, type of stoma and surgical technique. Furthermore, DC is frequently (60–70% of patients) asymptomatic and therefore overlooked. Symptoms may include pelvic discomfort, anorectal pain, tenesmus, mucous or bloody discharge, and are unrelated with severity of histologic changes. Interestingly, symptoms and inflammation are reversible after restoration of intestinal continuity [21, 22].

**Fig. 17:**
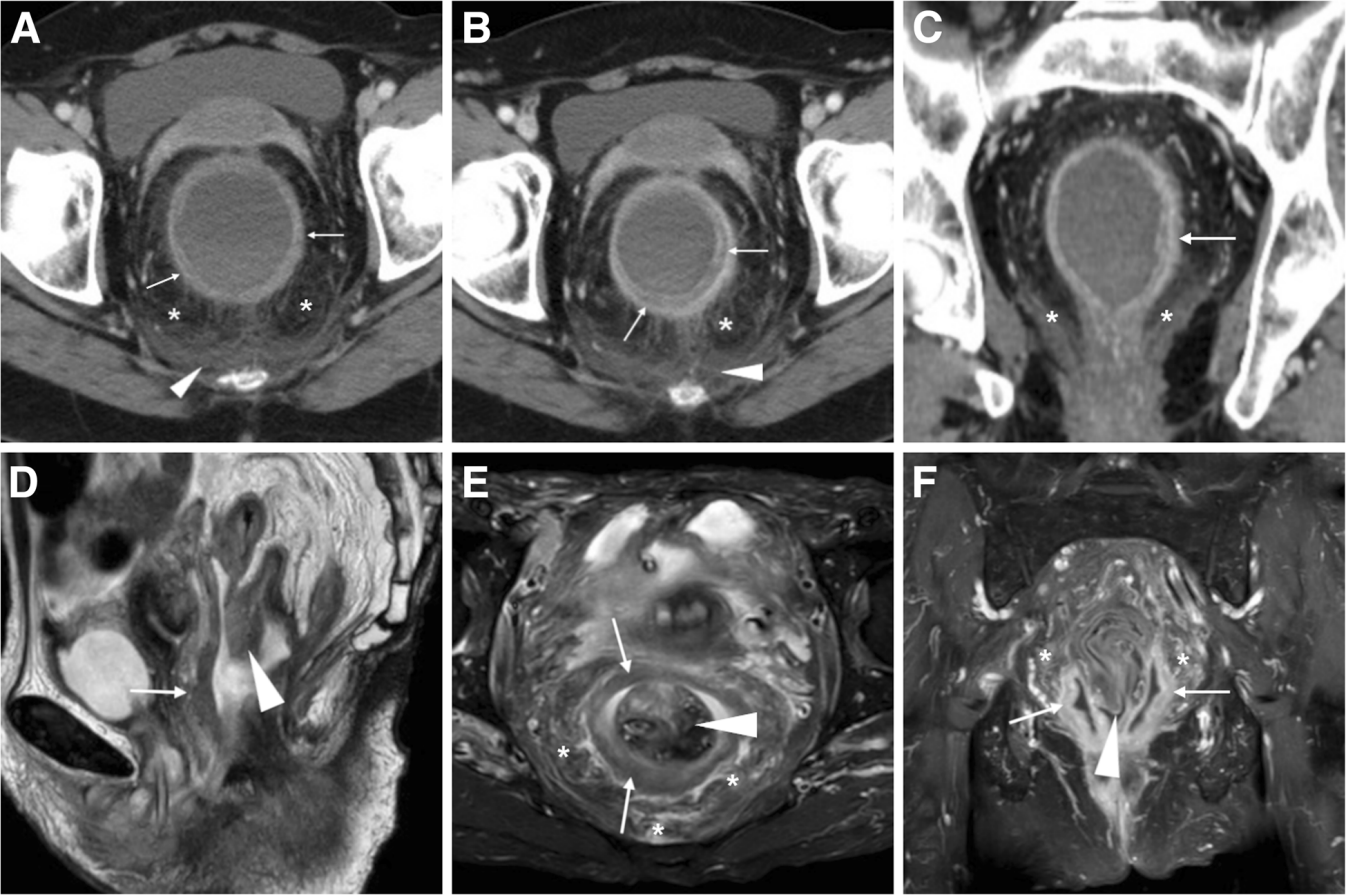
Two cases of diversion proctitis. **a**-**c**) a year after colectomy and terminal ileostomy for Crohn’s disease, initial CT (**a**) performed for perineal pain showed fluid-filled inflamed rectal stump with uniformly thick walls (thin arrows), mesorectal engorgement (*) and presacral fluid (arrowhead). Despite transanal drainage of fluid, repeated CT three months later (**b**-**c**) showed increased thickness and appearance of stratification at inflamed rectal walls (thin arrows). **d**-**f**) following colonic resection for indeterminate colitis, MRI including sagittal T2- (**a**), fat-suppressed axial T2- and post-gadolinium fat-suppressed T1-weighted (**C**) sequences showed markedly thickened oedematous and hypervascular rectal walls (thin arrows), perirectal fluid (*) and downwards invagination of the closed apex of rectal stump (arrowheads)

The diagnosis of diversion colitis relies on a combination of history, symptoms, endoscopic and histological findings. Infections such as pseudomembranous colitis are ruled out by negative stool cultures. Treatment consists in irrigation of the diverted colon with fatty-acids or butyrate enemas, or surgical reconstruction if feasible. At CT (Fig. 17A-C) diversion colitis should be suggested when nonspecific “proctocolitis” appearance are seen in the closed rectal stump in patients with a colostomy, including an increased wall thickness (over 3–4 mm) with stratified “target” or “water halo” appearance corresponding to inflamed mucosa, oedematous hypoenhancing submucosa [3, 23]. MRI even better shows mural oedema and perivisceral inflammatory changes (Fig. 17D–F). Unfortunately, these cross-sectional imaging closely resemble those of active IBD, therefore integration with clinical and endoscopic information is required when recanalisation is being considered in patients with underlying IBD [21, 22].

## Conclusion

Patients with ileostomies and colostomies are commonly encountered in Radiology departments, such as during perioperative hospitalisation following stoma creation or before recanalisation, or during cross-sectional imaging studies requested for follow-up of operated tumours or chronic IBD. When interpreting cross-sectional imaging studies, focused attention to the stoma site and awareness of expected appearances and of possible complications are required to avoid missing significant abnormalities. Additionally, specific imaging studies such as WSC-SE and combined CT plus WSC-SE may be helpful to provide surgeons the appropriate clinical information required to direct management.

## Abbreviations

APR: Abdomino-perineal resection
CRC: Colorectal cancer
CT: Computed tomography
DC: Diversion colitis
ECCO: European Crohn’s and Colitis Organization
EHS: European Hernia Society
IBD: Inflammatory bowel diseases
MRI: Magnetic resonance imaging
PEG: Polyethylenglycole
PH: Parastomal hernia
WSC-SE: Water-soluble contrast stomal enema
